# The first 90: Progress in HIV detection in Zhejiang Province, 2008–2018

**DOI:** 10.1371/journal.pone.0249517

**Published:** 2021-04-08

**Authors:** Lin Chen, Mingyu Luo, Yun Xu, Yan Xia, Xin Zhou, Wanjun Chen, Hui Wang, Tingting Jiang, Weiyong Chen, Yan Luo, Qiaoqin Ma, Jianmin Jiang, Xiaohong Pan

**Affiliations:** 1 Department of AIDS/STD Control and Prevention, Zhejiang Provincial Center for Disease Control, Hangzhou, Zhejiang, People’s Republic of China; 2 Department of AIDS/STD Control and Prevention, Hangzhou Center for Disease Control and Prevention, Hangzhou, Zhejiang, People’s Republic of China; Hebei Provincial Center for Disease Control and Prevention, CHINA

## Abstract

To analyze the results of HIV screening and the HIV-positive rate based on different HIV detection strategies in Zhejiang Province, China. Data were downloaded from the AIDS Prevention and Control Information System on May 1, 2019. HIV screening, prevalence, and incidence data were analyzed from 2008 to 2018. The incidence of HIV was calculated from the results of BED testing. SPSS software (ver. 19.0) was used for the analysis. The number of people screened for HIV increased by 229.7% from 2008 to 2018, while the incidence of HIV increased from 1.14‱ (2010) to 1.67‱ (2018), peak by 2015 (2.28‱). The proportion of people screened for HIV in medical institutions increased from 62.0% in 2008 to 67.1% in 2018, while of all positive tests, 47.9% were conducted at medical institutions in 2008, which increased to 63.2% in 2018. VCT and STD clinic attendees, who had only 4.5% of all those undergoing HIV tests, accounted for 23.7% of all HIV positive in 2018. **T**he rate of HIV-positive people and incidence of HIV both increased in Zhejiang Province between 2008 and 2015. The most effective strategy for detecting HIV new cases is screening visitors to VCT and STD clinics.

## Introduction

To prevent and control human immunodeficiency virus/acquired immunodeficiency syndrome (HIV/AIDS) worldwide, The Joint United Nations Programme on HIV/AIDS (UNAIDS) has set an ambitious 90-90-90 target for 2020: *i*.*e*., 90% of the people living with HIV (PLWH) know their status; 90% of the PLWH with known HIV status receive sustained antiretroviral therapy (ART); and 90% of the people receiving ART achieve viral suppression [1]. Remarkable progress has been made towards achieving this target globally; by 2016, 70% of the PLWH knew their status, 77% of these individuals were on ART, and 82% these were virally suppressed [2]. The highest levels of the three 90s are in western and central Europe, and the lowest are in western and central Africa [2–6]. Southeast Africa have made significant progression during the recent years [7]. In China, significant progress has been made and the three 90s were 68.9, 83.4, and 94.3%%, respectively [8]. Zhejiang Province is on the east coast of China and has a population of 56.6 million. Zhejiang has an average HIV prevalence of 0.05% and an estimated 28,520 PLWH in 2017 (based on the Spectrum model, unpublished). Zhejiang has been edging closer to the 90-90-90 target; according to the latest unreported data, 81.5% of PLWH know their status, 89.5% are on ART, and 92.6% of those being treated have suppressed viral loads. With the gap to reaching the first 90 narrowing, 18.5% of PLWH still do not know their status (unpublished data).

**Fig 1:**
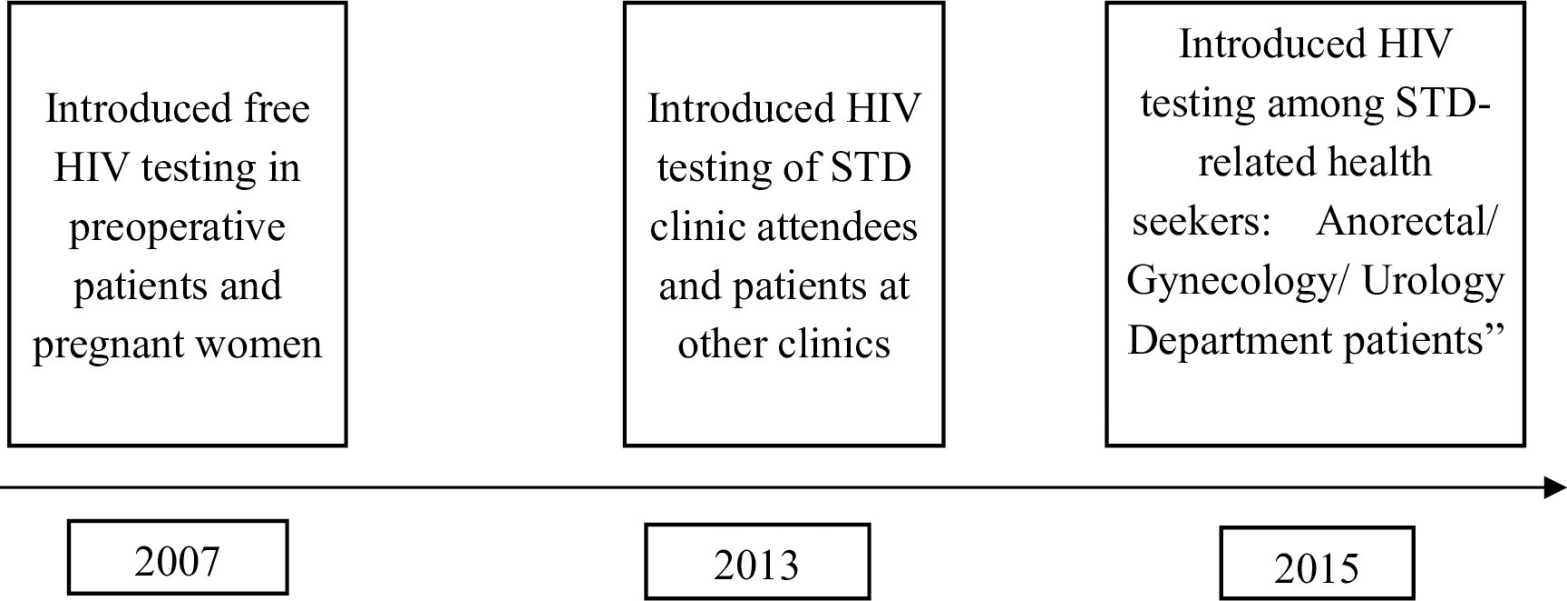
HIV screening strategy in medical institutions from 2007 to 2015. This fig shows the change of HIV screening strategy from 2007 to 2015. It helps the reader understand the results of this study.

To increase the accessibility of HIV testing, Zhejiang Province Center for Disease Control and Prevention (CDC) has conducted HIV screening in medical and non-medical institutions. Fig 1 shows the change in HIV screening strategy in medical institutions from 2007 to 2016. Another important strategy was establishing voluntary counseling and testing (VCT) clinics in the CDC and all large hospitals. The number of VCT clinics increased from 120 in 2008 to 330 in 2015. The screening strategy adopted was altered with the change in transmission mode, such as screening patients seeking treatment for sexually transmitted diseases (STDs) and responding to the HIV epidemic among men having sex with men (MSM) [9–15].

The incidence of HIV has been identified as the most precise indicator of ongoing HIV transmission, and also serves as an indicator of the efficacy of HIV interventions [16]. The IgG-capture BED enzyme immunoassay (BED-CEIA), developed by the US Centers for Disease Control and Prevention (CDC), was widely implemented in China between 2005 and 2014 to determine the incidence of HIV in this resource-limited country [17].

This paper analyzes the trend of HIV positive rate and HIV incidence rate, and the distribution in different detection pathways in Zhejiang Province between 2008 and 2015, exploring the optimal detection strategy and discussing on how we can reach the first 90 target.

## Materials and methods

### Data source

Data for the period 2008–2018were downloaded from the National AIDS Prevention and Control Information System on May 1, 2019. This information system was established by National Center for AIDS & STD Control and Prevention of China’s CDC. Form was unified designed including number of people screened for HIV, number of confirmed HIV positive, category of screening methods. The form was completed by all HIV primary screening testing agencies (CDC and medical institutions) every month and reviewed by local county, city and provincial CDC. Data on VCT was downloaded from national VCT Report System. VCT related-data was collected and uploaded by 341 certified agency including 94 CDC and 247 hospitals.

There were seven HIV screening strategies, including four used by medical institutions and three used by non-medical institutions. Fig 1 shows the three strategies used for HIV detection at medical institutions, *i*.*e*., examining preoperative patients, pregnant women, STD-related health seekers, and patients in other clinics. The three strategies used for HIV screening at non-medical institutions included VCT, and screening of high-risk people and those undergoing medical check-ups. High-risk people are those who are vulnerable or show risky behavior, such as the spouses and offspring of HIV-positive patients, those with occupational exposure, entertainers, paid blood donors, detainees, and persons subjected to re-education. The medical check-ups included premarital examinations, personnel entry and exit physical examinations, and examinations of military recruits and blood donors.

### Laboratory testing

From patients diagnosed as HIV-positive by confirmatory Western blot (WB) testing at the local CDC, 3~5 mL of venous blood was collected and transported to the HIV/AIDS library of the Zhejiang provincial CDC. Antibody-positive specimens on WB were tested to identify recent HIV infection (RHI) using BED-CEIA (Calypte Biomedical, Portland, OR, USA). Specimens from unambiguous previously diagnosed cased were excluded from the analysis; these included AIDS patients, patients receiving antiretroviral therapy, and cases diagnosed more than 6 months previously.

The BED assay was performed by the Zhejiang provincial CDC according to the manufacturer’s instructions. The calibrator ratio (ODn; specimen OD/calibrator OD) was used to normalize the optical density (OD) of test specimens to minimize internal variation. Samples with ODn ≤ 1.2 were tested again in triplicate and the median values were used for evaluation. Samples with ODn ≤ 0.8 were considered to be recent infections, suggesting that seroconversion occurred within the previous 168 days (95% CI, 155–184 days) [17].

### Statistical analysis

The statistical analyses is performed using Excel (ver. 3.0; Microsoft Corp, Redmond, WA, USA), SPSS (ver. 19.0; IBM Corp., Armonk, NY, USA) and SAS (version 9.2; SAS Institute, Cary, NC, USA). Excel is used to draw the figures. The Cochran-Armitage Trend Test is used to compare categorical variables for the period 2008–2018. We calculate the incidence of HIV based on the BED-CEIA approach, which express as the number of new infections in the last 6 months. We estimate the incidence of HIV using Eq (1) [18,19]:

I(%)=[(365/w)*R]/[N+(365/w)*R/2]*100% Eq (1)

$$95\%\mathrm{CI}=1\pm 1.96\left(\frac{1}{\sqrt{R}}\right)$$

Where N is the number of HIV-1-negative cases, R is the number of BED-CEIA-positive cases, and W is the testing window (168 days in China [20]).

We use a Microsoft Excel-based program to calculate the BED-estimated HIV incidence. Significance was determined at the *p*≤0.05 level, and all hypothesis tests were two-sided.

### Ethics approval and informed consent

This study was reviewed and approved by the Institutional Review Board of the National Center for AIDS/STD Control and Prevention, Chinese center for disease control and prevention (IRB approval number: X120331209). Data in this research were from National AIDS Prevention and Control Information System and from routine work. There was no privacy information on patients. Informed consent was not needed in this study.

## Results

### Trends in HIV testing, prevalence, and incidence

**Fig 2:**
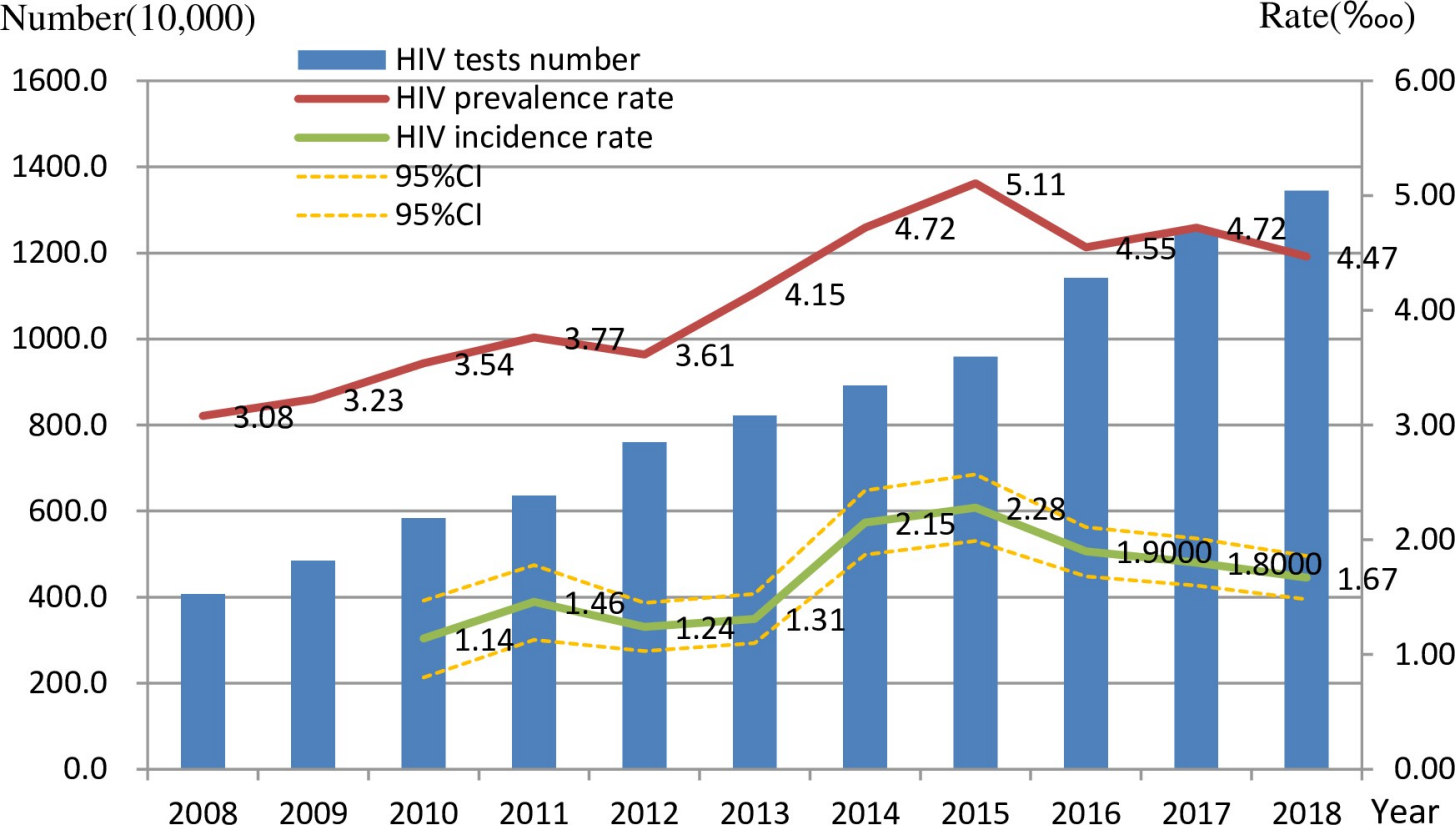
Number of HIV testing, rate of HIV-positive and HIV incidence rate in Zhejiang province between 2008 to 2018. This figure shows the change of HIV testing number, HIV prevalence and HIV incidence in whole population.

During this 11-year study, the amount of people screened for HIV in Zhejiang Province increased markedly, by 229.7% from 2008 to 2018. The HIV-positive rate increased from 3.08‱ (2008) to 3.54‱ (2010), 3.61‱ (2012), and 5.11‱ (2015),4.47‱ (2018), for an overall increase of 45.1‱. The incidence of HIV increased by 1.14, 1.24, 2.28 and 1.67‱ for the years 2010, 2012, 2015 and 2018, respectively (Fig 2). The HIV positive rate and HIV incidence rate were both peak by 2015.

According to the regional distribution, the HIV incidence rate in Hangzhou and Jinhua were higher than that in other 5 cities (Shaoxing, Ningbo, Quzhou, Zhoushan and Huzhou). Ningbo city was identified as high HIV-positive rate (5.67‱) and low HIV incidence rate (1.24‱) (Table 1)

**Table 1:** HIV testing, HIV prevalence, HIV incidence rate in 11 cities in Zhejiang province, 2018.

City	HIV tests (10,000)	HIV prevalence rate (‱)	HIV incidence rate (‱,95%CI)
Jinhua	156.0	5.14	2.50 (1.81–3.19)
Hangzhou	285.9	5.19	2.34 (1.84–2.84)
Lishui	55.0	2.82	1.85 (0.88–2.82)
Wenzhou	210.6	3.94	1.64 (1.15–2.12)
Jiaxin	101.0	4.31	1.42 (0.82–2.02)
Taizhou	121.9	4.86	1.27 (0.69–1.85)
Shaoxing	107.0	3.44	1.24 (0.68–1.80)
Ningbo	162.4	5.67	1.24 (0.75–1.72)
Quzhou	43.6	2.98	0.91 (0.20–1.63)
Zhoushan	26.1	2.61	0.82 (0.00–1.69)
Huzhou	75.2	3.09	0.73 (0.19–1.27)
Total	1344.7	4.47	1.67 (1.48–1.86)

This show the difference of HIV prevalence, HIV incidence in different cities.

### HIV testing and incidence rates in medical and non-medical institutions

The proportion of people screened for HIV in medical institutions increased from 62.0% in 2008 to 67.1% in 2018, while of all positive tests, 47.9% were conducted at medical institutions in 2008, which increased to 63.2% in 2018. In 2018, the top three groups of individuals screened were preoperative patients, the high-risk population, and other patients, accounting for 36.9% (4,989,454/13,513,480), 19.8% (2,676,179 /13,513,480) and 19.4% (2,618,238/13,513,480) of those tested, respectively.

In medical institutions, the number of HIV tests performed increased by 330.8%, 273.4%, 116.3%, and 608.5% from 2008 to 2018 for STD clinic attendees, preoperative patients, pregnant women, and other patients, respectively. The chi-square test for trend showed that the increases in the HIV-positive rate in all four groups were significant (all *p*<0.01), except for the incidence of HIV among other patients (Table 2). Compared with HIV incidence rate in 2008, the incidence rates were higher after 2012 among STD clinic attendees and preoperative patients. In 2018, STD clinic attendees had the highest HIV-positive (13.76‱) and HIV incidence (6.78‱) rates, followed by other patients (5.34‱ and 3.08‱, respectively) (Table 2).

**Table 2:** Number and HIV screening, HIV positive rate and HIV incidence rate among population in medical institution.

	2008	2010	2012	2014	2016	2018	Trend χ^2^	*p*
STD clinic attendee								
HIV-testing number (N)	119452	169221	206095	276618	400486	514579		
HIV-positive rate (‱)	7.62	8.33	9.70	16.12	15.66	13.76	63.91	<0.001
HIV incidence rate (‱)	-	-	2.45(1.10–3.80)	7.12(4.91–9.32)	7.35(4.97–9.72)	6.78(3.86–9.71)		
Pre-operation patients								
HIV-testing number (N)	1336369	2000022	2795021	3503198	4197953	4989454		
HIV-positive rate (‱)	2.00	2.27	2.40	3.02	3.00	3.19	93.78	<0.001
HIV incidence rate (‱)	-	-	0.58(0.29–0.87)	1.39(1.01–1.77)	1.14(0.81–1.48)	0.96(0.49–1.43)		
Pregnant women								
HIV-testing number (N)	701050	937228	1261469	1394574	1600791	1516569		
HIV-positive rate (‱)	0.86	0.65	0.58	0.53	0.55	0.31	24.89	<0.01
HIV incidence rate (‱)	-	-	0.15(0.01–0.29)	0.21(0–0.43)	0.18(0–0.38)	0.34(0–0.84)		
Other patients								
HIV-testing number (N)	369522	615368	854986	1295661	1818095	2618238		
HIV-positive rate (‱)	4.95	5.95	5.35	6.02	6.44	5.34	0.005	0.943
HIV incidence rate (‱)	-	-	1.18(0.70–1.65)	2.14(1.60–2.68)	2.05(1.55–2.55)	3.08(2.02–4.13)		

Note: Other patients refers to patients other than STD clinic attendee, pre-operation patient, pregnant women in medical institution.

The table shows HIV screening number, HIV positive rate and HIV incidence rate among population in different clinics of medical institution.

In non-medical institutions, the number of HIV testing from 2008 to 2018 increased by 23.5%,250.0%, and 55.4% in the VCT, high-risk people, and medical examination groups, respectively. Increased HIV-positive rates were seen in the VCT group and high risk people. In 2018, the VCT group had the highest HIV-positive rate (120.02‱) and incidence of HIV (70.57‱), followed by high-risk people (3.82‱ and 2.07%, respectively). HIV incidence rate in 2012 was 34.80‱, lower than that in 2014, 2016 and 2018 among VCT clinic counselor (Table 3).

**Table 3:** Number and HIV screening, HIV positive rate and HIV incidence rate among population in non-medical institution.

	2008	2010	2012	2014	2016	2018	Trend χ^2^	*p*
VCT								
HIV-testing number (N)	75927	70706	93988	96706	94614	93733		
HIV-positive rate (‱)	25.81	47.52	61.50	99.68	111.72	120.02	736.12	<0.001
HIV incidence rate (‱)	-	-	34.80(24.99–44.60)	57.39(44.58–70.21)	62.27(49.50–75.04)	70.57(50.69–90.44)		
high risk people								
HIV-testing number (N)	764706	961089	1239693	1220994	2147501	2676179		
HIV-positive rate (‱)	4.96	5.733	4.56	5.68	3.921	3.82	64.034	<0.001
HIV incidence rate (‱)	-	-	1.96(1.37–2.55)	2.50(1.91–3.09)	1.46(1.04–1.87)	2.07(1.42–2.72)		
medical examination People								
HIV-testing number (N)	710804	1084398	1152443	1133921	1259412	1104672		
HIV-positive rate (‱)	1.39	1.11	1.76	1.70	1.42	1.14	0.273	0.601
HIV incidence rate (‱)	-	-	0.47(0.17–0.77)	0.72(0.37–1.06)	0.61(0.28–0.94)	1.12(0.27–1.98)		

Note: 1.VCT: Voluntary counseling and testing.

2. Data from National AIDS Prevention and Control Information System.

It shows the distribution of HIV positive rate and HIV incidence rate in different kind of non-medical institution which was compared with Table 2. Tables 2 and 3 was made to be compared and to shows the effective of different screening strategy in Fig 1.

Table 4 shows the numbers of people screened for HIV and the percentage testing positive in the CDC and hospitals from 2012 to 2018. There was a 3.5% decrease in the number of HIV tests in hospital and a 24.0% decrease at the CDC, over the study period. The percentage of people testing positive for HIV increased continually, being 143.27, 229.50, 275.84 and 328.13‱ in 2012, 2014, 2016 and 2018, respectively (trend chi-square = 328.2, *p*<0.01). There was no significant change in the percentage of people testing positive for HIV in hospitals (Table 4).

**Table 4:** Number and rate of HIV screening-test among population in VCT.

Year	CDC			Hospital		
	HIV testing (N)	Screening-Positive (N)	Screening-Positive rate (‱)	HIV testing (N)	Screening-Positive (N)	Screening-Positive rate (‱)
2012	47324	678	143.27	52905	161	30.43
2014	45141	1036	229.50	54175	122	22.52
2016	40929	1129	275.84	54684	137	25.05
2018	35961	1180	328.13	51066	129	25.26
Trend χ^2^	328.200			1.725		
*p*	<0.001			0.189		

Note: Data from National VCT Report System.

It shows the distribution of HIV testing number and HIV positive rate in different institution of VCT clinics.

## Discussion

This study found that remarkable progress has been made between 2008–2018 in achieving the first 90 HIV target, with a more than 100% increase in HIV tests performed and a 45.1% increase in HIV-positive results, of which almost 50% were new infections. However, the increased percentage of positive tests could be a result of three different situations: increased incidence, increased number of people taking a HIV test or both of the above. Health care providers, such as community-based organizations (CBO), public hospital and CDC did a better job for detecting HIV positives over time. During the last decade, Zhejiang Province has made great efforts toward improving HIV detection, including screening of preoperative patients and pregnant women, and especially of VCT and STD-related health seekers. The cities of Hangzhou, Wenzhou conducted the most HIV screening tests and had high HIV-positive and incidence rates, because both cities have top-class hospitals and key populations are present there [18].

In particular, STD and VCT clinics play important roles in HIV detection [16–18]. We found that the HIV-positive rate and incidence were higher among STD clinic patients and those undergoing VCT than in other populations. Nevertheless, VCT in hospitals plays a much smaller role in HIV detection compared with VCT at the CDC, perhaps because hospitals have less space and fewer doctors for HIV counseling and there is more advertising for VCT in CDCs, especially for MSM [21]. The HIV-positive and incidence rates were almost 20 times higher among STD clinic patients than in high-risk people and those undergoing medical examinations. This is the result of a provider-initiated HIV testing strategy implemented in China in 2009. STD clinic patients are also more likely to contract HIV infection because of high-risk behavior [22]. However, the number of HIV tests did not increase as rapidly as in other hospital clinics. Traynor *et al*. revealed that 46.2% of STD clinic patients were not tested for HIV in the previous 12 months and 74.1% missed an opportunity for HIV testing in an STD clinic [23].

In our study, in 2018, 67.1% of those screened for HIV were tested in medical institutions, compared with 32.9% in non-medical institutions; meanwhile, the proportions of HIV-positive cases were 63.2% and 36.8%, respectively. Therefore, HIV screening in non-medical institutions is more effective in finding positives. However, medical institutions play a key role in increasing HIV detection rates because many people access them, including vulnerable and low-risk people. Another reason for supporting medical institution screening strategies is that they are easy to implement. The main problem for medical institutions is how to catch vulnerable people and implement interventions, as is done with STD patients.

Compared with HIV screening strategies other than those implemented at STD clinics and CDCs, screening of preoperative patients tested considerably more people and yielded a low HIV incidence rate. Pregnant women had the lowest HIV-positive rate. Screening at preoperative patients and pregnant women was originally designed to protect doctors and infants, and was not the most effective strategy. Although the HIV-positive rates were low in these two groups, many HIV-positive patients were detected, demonstrating the importance of such screening. The strategy of screening high-risk people, including MSM, IDU, and CSW, for HIV was more effective.

The overall incidence of HIV in this study was 1.67‱ in 2018. The incidence of HIV was highest in the VCT clinical subgroup, at 70.57‱, which was much lower than in Sichuan, Yunnan, and Jiangsu Provinces, but higher than that in 2008 [24–27]. The main reason for this is that HIV infection in MSM has increased rapidly in the 7 years since 2011 [28,29]. Approximately 14.1% (95% CI = 10.1–19.2%) of MSM perceived a risk of contracting HIV and 48.1% had never been tested for HIV; this suggests that HIV transmission will increase without early testing [30].

There were limitations to this study. First, the assessment of the effect of HIV policy on HIV detection did not involve a rigorous, randomized pre- and post-test design. The increase in the number of HIV tests performed is not due solely to policy, such as implementation of new programs. Another limitation was that the data from different source were inconsistent regarding VCT. Despite these limitations, we are confident that the large amount of data will eliminate the difference between the two databases and reveal the extent of the HIV epidemic and effects of HIV testing.

## Conclusion

With the increase in HIV screening, the HIV-positive and incidence rates increased simultaneously in Zhejiang Province between 2008 and 2018. The highest HIV-positive and incidence rates were in the cities Hangzhou and Ningbo. Compared with non-medical institutions, tests conducted at medical institutions showed a lower HIV-positive rate and incidence of HIV, but detected more HIV-positive patients. The highest incidence of HIV was reported by VCT and STD clinics, where plays an important role in detecting new cases of HIV infection. The best strategy for increasing the rate of detection of HIV cases is one that increases HIV screening in both medical and non-medical institutions, especially of key populations and clients of VCT and STD clinics.
